# [Retracted] miR‑25 promotes metastasis via targeting FBXW7 in esophageal squamous cell carcinoma

**DOI:** 10.3892/or.2023.8644

**Published:** 2023-10-09

**Authors:** Ye Hua, Kai Zhao, Gang Tao, Chunhua Dai, Yuting Su

Oncol Rep 38: 3030–3038, 2017; DOI: 10.3892/or.2017.5995

Following the publication of the above paper, it was drawn to the Editor's attention by a concerned reader that a large number of data panels showing cell migration and invasion assay data in Figs. 3C and 5 contained overlapping sections, such that data that were intended to show results obtained under different experimental conditions may have been derived from a smaller number of original sources. In addition, certain of the data in this pair of figures were strikingly similar to data that were submitted for publication in another journal at around the same time as the above paper was submitted to *Oncology Reports*. Finally, regarding the western blotting data shown in Fig. 4B, an obvious splice in the gel strip was noticed for the FBXW7 bands, whereas no equivalent splice was present in the associated GADPH loading control, suggesting that these data originated from different gels.

Owing to the fact that the contentious data in the above article were under consideration for publication at around the time that this was submitted to *Oncology Reports*, in addition the other features of concern regarding the data, the Editor has decided that this paper should be retracted from the Journal on account of a lack of confidence in the presented data. The authors were asked for an explanation to account for these concerns, but the Editorial Office did not receive a reply. The Editor apologizes to the readership for any inconvenience caused.

